# The Association between Blunt Cardiac Injury and Isolated Sternal Fracture

**DOI:** 10.1155/2014/629687

**Published:** 2014-02-06

**Authors:** Anahita Dua, Jason McMaster, Pathik J. Desai, Sapan S. Desai, SreyRam Kuy, Maggy Mata, Jamie Cooper

**Affiliations:** ^1^Center for Translational Injury Research (CeTIR), Department of Surgery, University of Texas-Houston, 6431 Fannin Street, Houston, TX 77030, USA; ^2^Department of Emergency Medicine, Aberdeen Royal Infirmary, University of Aberdeen School of Medicine, Aberdeen AB25 2ZN, UK; ^3^Department of Surgery, Medical College of Wisconsin, Milwaukee, WI 53226, USA; ^4^Department of Medicine, Medical University of Lublin, 20 080 Lublin, Poland; ^5^Department of Surgery, Duke University Medical Center, Durham, NC 27710, USA

## Abstract

The treatment of isolated sternal fractures (ISF) throughout the world is heterogeneous. This study aimed to identify the incidence, morbidity, and mortality associated with isolated fractures of the sternum and describe current practice for diagnosis and management of ISF and cardiac injury at a level I trauma center in the UK. A retrospective cohort study of adult patients (>16 years) with ISF presenting from 2006 to 2010 was conducted. Eighty-eight patients with ISF were identified. Most patients (88%, 77) were admitted to hospital with 66% (58) of them discharged within 48 hours. Two (2%) patients had an ER EKG with abnormality but both resolved to normal sinus rhythm within 6 hours of follow-up. Serum CEs were drawn from 55 (63%) patients with only 2 (2%) having a rise in serum troponin >0.04; however, in both of these patients troponin quickly normalized. Six (7%) patients underwent echocardiograms without significant findings. In all 88 patients with ISF, no cases of clinically significant cardiac injury were identified. Patients presenting with an isolated sternal fracture with no changes on EKG or chest X-ray do not warrant an admission to hospital and may be discharged from the ER.

## 1. Introduction

Sternal fractures were once thought to be high-morbidity injuries given the proximity to the myocardium and likelihood of associated injury [1]. Caution is appropriately warranted when evaluating and treating patients with this injury as sternal fracture from motor vehicle crashes (MVC) has a 1.5% incidence of cardiac dysrhythmia requiring treatment and a mortality rate of up to 1% if not managed appropriately [1].

Isolated sternal fracture may be defined as a sternal fracture without any other thoracic injury such as rib fracture, hemothorax, or pneumothorax. The incidence of isolated sternal fractures (ISFs) is rising given the increasing use of seatbelts since legislation was implemented in the UK in 1983 and a standardized, safe protocol detailing the management of this condition may reduce bed occupancy and might result in financial savings. Some studies have concluded that rates of morbidity are low and ER discharge may be appropriate after sternal fracture while others have concluded that sternal fractures and left-sided serial rib fractures are predictive of cardiac injury and warrant monitoring [1–3].

As a result of these conflicting reports, the treatment of isolated sternal fractures (ISFs) throughout the world is heterogeneous ranging from ER discharge with no follow-up to admission to the cardiothoracic unit for monitoring and analgesia. Traumatic isolated sternal fracture (ISF) has traditionally mandated hospital admission with investigation for blunt cardiac injury involving repeated cardiac enzyme (CE) studies every 6 hours, invasive testing, and even cardiothoracic unit admission in the UK. Recent studies have commented on the poor clinical yield of this approach and suggested that in patients with a normal EKG, benign chest X-ray (CXR), and stable hemodynamic parameters, significant cardiac injury is unlikely and post-ED discharge could be recommended [2]. Even fewer studies have detailed what the resultant clinical sequelae are in patients with abnormal initial CXR, CE, or EKG on admission.

This study aims to identify the incidence, morbidity, and mortality associated with isolated fractures of the sternum and describe current practice for diagnosis and management of ISF and cardiac injury at a large, level I trauma center in the UK.

## 2. Methods

A retrospective review of the medical records of blunt trauma patients with sternal fracture admitted to a UK level one trauma center from October 2006 to March 2010 was undertaken to determine the relationship between sternal fractures and clinically significant myocardial injury and to assess the usefulness of cardiac evaluation and monitoring in these patients. Patients with any concomitant injuries aside from minor soft tissue lacerations were excluded.

Age, sex, and mechanism of injury along with the initial physiological parameters and investigative parameters such as EKGs, CXRs, and CEs involving Troponin >0.04 were documented for statistical analysis.

Data analysis was completed using SigmaStat 3.5 which defined parameters of consideration in the presence of clinical cardiovascular deterioration indicated by hypotension, cardiac arrhythmia, and elevated CEs or if the echocardiogram demonstrated significant wall motion abnormality illustrating significant myocardial trauma. Main outcome measures included definitions, morbidity, and mortality.

## 3. Results

Overall, 127 patients presented with documented isolated sternal fracture from October 2006 to March 2010. Eight patients were incorrectly identified after review of their CXR and were excluded. Thirty-one patients had missing data in their medical records and were excluded. Overall, 88 patients between the ages of 17 and 92 years (66% female, 34% male) were identified for inclusion in this study.

Among the 88 patients identified, 66% were female and 34% were male with an age range of 17–92 years. Motor vehicle crash (MVC) accounted for 72 (82%) cases and ISFs were displaced in 10 (11%) instances. The majority of patients (88%, 77) were admitted to hospital with 66% (58) of them discharged within 48 hours. Two (2%) patients had an ER EKG with abnormality (patients had unexplained ST changes) but both resolved to normal sinus rhythm within 6-hour repeat EKG follow-up. A single patient (1%) was presented with a widened mediastinum on CXR but CT imaging excluded aortic injury. Serum CEs were drawn in 55 (63%) patients with only 2 (2%) having a rise in serum troponin >0.04; however, in both of these patients troponin quickly normalized and neither suffered any cardiac complications. Six (7%) patients underwent echocardiograms all without significant findings. In all 88 patients with ISF, no cases of clinically significant cardiac injury were identified. All patients were followed up in clinic 1 week after injury or hospital discharge.

## 4. Discussion

Sternal fractures account for 8% of admissions for thoracic trauma with motor vehicle collisions accounting for 60–90% of sternal fractures [1–3]. The mortality rate from isolated sternal fracture is low. Morbidity and mortality result primarily in aortic disruption, cardiac contusions, and pulmonary contusions in isolated sternal fractures [1, 2].

The most common complication after an injury in patients with isolated sternal fractures is pain; up to two-thirds of patients require management with analgesia alone. However, long term complications such as painful pseudarthrosis may develop requiring surgical correction. In those patients with displaced or unstable sternal fractures, operative fixation with sternal wires or formal osteosynthesis using plate and screws may be necessary. Not intervening surgically can cause debilitating chest pain associated with flail chest and compromised pulmonary or cardiac function. Morphine and nonsteroidal based analgesia may prove inadequate in this patient cohort. Duncan and colleagues published a report on the use of a continuous infusion of local anesthetic and opioid to the sternal fracture site via a periosteally positioned catheter. This technique was successful but may not be available in all facilities. Pain control is especially important given the association with respiratory compromise if uncontrolled. Elderly persons with chest wall fractures are at increased risk of atelectasis and may be admitted for pain control in order to prevent respiratory complications.

Potaris et al. [2] reported a morality rate of 0.8% [2]. Other reports reported that there is no true relationship between sternal fractures and blunt cardiac trauma; hence sternal fracture is not a marker for clinically significant myocardial injury [4, 5]. Symptoms can vary considerably but most patients report localized sternal pain. Dyspnea is present in 15–20% of these patients and may indicate associated cardiopulmonary contusion. If a dysrhythmia has occurred, palpitations may be apparent but these may be present as results of the pain or stress of the injury.

The development of an associated myocardial contusion is dependent on a number of factors including the type of sternal fracture and CXR with a widened mediastinum. von Garrel et al [6] noted that after traffic accidents patients are more likely to sustain a non-displaced or slightly displaced sternal fracture (75.5%) than a severely displaced fracture (24.5%).

Rashid et al. [7] reported that retrosternal hematomas were found adjacent to many fractures and ranged in size from a few mm to 2 cm but were most common in fractures of the body of sternum. However, this had no correlation with cardiac injury. Conversely, patients with a widened mediastinum had a higher injury severity score, longer hospital stay, and more associated cardiac lesions. Overall, in keeping with our data, Rashid and colleagues reported that no serious cardiac or aortic injuries were detected in their series of 418 patients either [7].

Over the last 20 years, studies have detailed their experience with isolated sternal fractures; not a single one of these studies had any cardiac complications or death from cardiac injury due to an ISF [8–12]. Aside from noting a widened mediastinum, other reports have not been clear regarding the correct investigations to determine which patients require cardiac monitoring. Drs. Heyes and Vincent [13] noted that ECG results were not very specific but were sensitive enough in identifying potential problems. Fabian et al. [14] deduced that the CE level would be the most relevant test in determining myocardial injury but another study by Drs. Peer and Firmin [12] concluded that only ECG abnormalities could accurately identify myocardial trauma sites and hence CEs and echocardiogram were not useful in determining which patient required further care [12].

Follow-up in the outpatient setting is important to monitor healing and ensure adequate pain control; however in patients with sternal fractures, no specific diagnostic or intervention based management strategies are required at follow-up unless indicated by patient symptoms.

## 5. Conclusion

Our results suggest that patients sustaining ISF, even the displaced type, could be categorized as unlikely to develop cardiac complications. In the absence of clinical evidence regarding cardiac or respiratory complications, patients were discharged after ER, barring any evidence of physiological compromise, ECG irregularity, or CXR abnormality. However, the role for CEs remains controversial and further studies are warranted to establish an acceptable guideline for the management of these patients.
